# Randomised trial of proton vs. carbon ion radiation therapy in patients with chordoma of the skull base, clinical phase III study HIT-1-Study

**DOI:** 10.1186/1471-2407-10-607

**Published:** 2010-11-05

**Authors:** Anna V Nikoghosyan, Irini Karapanagiotou-Schenkel, Marc W Münter, Alexandra D Jensen, Stephanie E Combs, Jürgen Debus

**Affiliations:** 1 Dept. of Clinical Radiology, University of Heidelberg, INF 400, 69120 Heidelberg, Germany; 2 Biometrics and Data Management, DKFZ G040, Patient and NCT Clinical Trial Center, Im Neuenheimer Feld 581, 69120 Heidelberg, Germany

## Abstract

**Background:**

Chordomas of the skull base are relative rare lesions of the bones. Surgical resection is the primary treatment standard, though complete resection is nearly impossible due to close proximity to critical and hence also dose limiting organs for radiation therapy. Level of recurrence after surgery alone is comparatively high, so adjuvant radiation therapy is very important for the improvement of local control rates. Proton therapy is the gold standard in the treatment of skull base chordomas. However, high-LET beams such as carbon ions theoretically offer biologic advantages by enhanced biologic effectiveness in slow-growing tumors.

**Methods/design:**

This clinical study is a prospective randomised phase III trial. The trial will be carried out at Heidelberger Ionenstrahl-Therapie centre (HIT) and is a monocentric study.

Patients with skull base chordoma will be randomised to either proton or carbon ion radiation therapy. As a standard, patients will undergo non-invasive, rigid immobilization and target volume delineation will be carried out based on CT and MRI data. The biologically isoeffective target dose to the PTV in carbon ion treatment (accelerated dose) will be 63 Gy E ± 5% and 72 Gy E ± 5% (standard dose) in proton therapy respectively. Local-progression free survival (LPFS) will be analysed as primary end point. Toxicity and overall survival are the secondary end points. Additional examined parameters are patterns of recurrence, prognostic factors and plan quality analysis.

**Discussion:**

Up until now it was impossible to compare two different particle therapies, i.e. protons and carbon ions directly at the same facility.

The aim of this study is to find out, whether the biological advantages of carbon ion therapy can also be clinically confirmed and translated into the better local control rates in the treatment of skull base chordomas.

**Trial registration:**

ClinicalTrials.gov identifier: NCT01182779

## Background

Chordomas (1 - 4% of all malignant bone tumors) of the skull base are relative rare lesions of the bones. The incidence is around 100 new cases per year in Germany and 0.08/100000 in the US corresponding to around 300 new cases per year [1]. Chordomas arise from embryonic remnants of the notochord and are found at the base of the skull area in 35% of all cases. According to the histopathological findings, chordomas are divided into conventional (most common), chondroid and dedifferentiated types [2,3]. Histological differentiation between chordomas and chondrosarcomas is difficult and must contain immunhistochemical tests [4]. Chordoma is immunopositive for epithelial markers like cytokeratin and endothelial membrane antigen (EMA), whereas chondrosarcoma is negative for both. Both chordomas and chondrosarcomas can be positive for S-100 and vimentin.

The average age at the diagnosis is 49 years for base of skull localization. Also, the base of the skull is the most common localisation of chordomas in children and adolescents. In children and adolescents chordomas may behave more aggressively [5]. Men are affected more frequently than women.

Surgical resection is the primary treatment standard, though complete resection is nearly impossible due to close proximity to critical and hence also dose limiting organs for radiation therapy i.e. optic nerves, chiasm and brainstem. Level of recurrence after surgery alone is with reported rates between 50% and 100% [6] comparatively high, so adjuvant radiation therapy is very important for the improvement of local control rates in the primary treatment even after complete resection. Tumour volume is an important prognostic factor, hence a tumour debulking is usually required before radiation therapy application [7]. Other prognostic relevant risk factors are the histological tumour type, resection status, gender, and the age of the patient [8,9].

Radiation resistance is a common characteristic of chordomas [10,11], so high total doses are needed to achive acceptable local control rates after radiotherapy. Conformal precision and image guided radiation therapy techniques provide a safe technique for dose escalation [12]. The physical characteristics of carbon ions or protons such as inverted dose profile allow steep dose gradients and therefore provide further benefit in this field by potentially reducing toxicity.

So far, proton therapy is the gold standard in treatment of rare skull-base tumours like chordoma and low grade chondrosarcoma. The Loma Linda University Medical Center (LLUMC) [13] and the Massachusetts General Hospital (MGH) in Boston [14] have the largest experience in particle therapy for these entities. 3-year local control for chordomas after fractionated proton radiation therapy in 33 patients at LLUMC was 67% and the actuarial 5 year survival rate was 79% respectively [14]. The outcome of 519 cases of chordomas and chondrosarcomas of the skull base including 290 chordomas treated with a combination of proton and photon therapy at MGH/HCL shows a significant difference in local control and survival rates between the patients with chordoma and chondrosarcoma. 5- and 10-year local progression free survival was 98% and 94% for chondrosarcomas and 73% and 54% for chordomas [13,14].

Proton therapy results from PSI in Villingen, Switzerland were published by Weber et al. and showed 3 year local control rates of 87.5% for chordomas. However, the treated tumour volumes with a median GTV of 16.4 ml were relatively small. 29 patients, among them 18 patients with chordoma were treated to a median target dose of 74 GyE. The 3-year actuarial PFS rate for the entire patient cohort was 90% [15].

Carbon ions though, have a higher biological effectiveness than either protons or photons, which might improve the results of these radio-resistant tumours [16]. Carbon ion therapy is available only at the National Institute of Radiological Sciences (NIRS) in Japan and up to July 2008 has been available at the Gesellschaft für Schwerionenforschung (GSI) Darmstadt in Germany. The experience of our Japanese colleagues is limited to 40 patients with chordoma and chondrosarcoma of the skull base who could be treated effectively and without serious side effects [17]. NIRS beam delivery technique relies on passive scanning necessitating various modulators to adjust for treatment depth and tissue inhomogeneities within the beam path.

**Figure 1:**
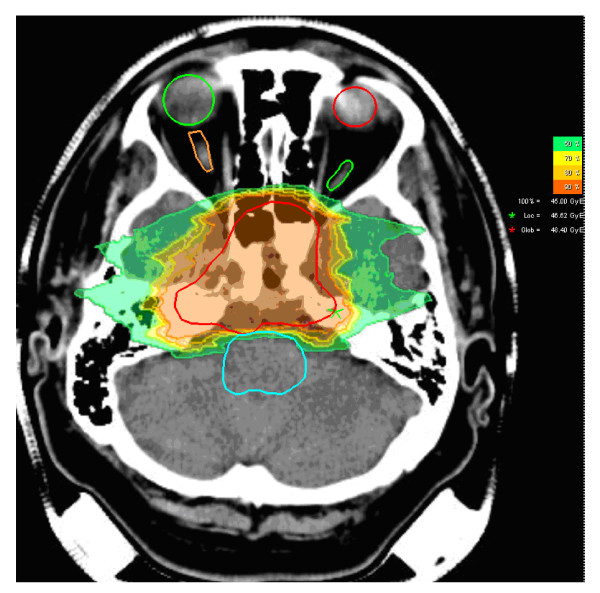
**Typical dose distribution of carbon ion therapy by clivus chordoma patient (axial view and dose legend; red line defines CTV; both eyes, optic nerves and brain steam are also shown)**.

In comparison to the Japanese centres, the facility at GSI as well as the Heidelberger Ionenstrahl-Therapie centre (HIT) relies on active beam delivery using the raster-scan technique. In prior series, each treatment plan was biologically optimized at GSI as well as at HIT (Figure 1). About 300 patients with base of skull chordomas and chondrosarcomas have been treated so far. Initially, these patients were treated within a clinical Phase I/II study. This study was able to demonstrate excellent clinical results and carbon ion therapy became approved as the best therapy available in Germany. The actuarial local control rates for the chordoma patients after 3 and 5 years were 80.6% and 70%, the 3 and 5 year overall survival rates were 91.8% and 88.5%, respectively [18].

## Methods and Design

### Primary objectives/endpoints of the study

The primary objective of this study is to evaluate, if the innovative therapy (carbon ion irradiation) in chordomas is superior to the standard proton treatment with respect to the Local-progression free survival (LPFS) defined as time from the randomisation to observed local recurrence or death from any cause in the absence of documented local disease progression. Local recurrence defined as MRT or CT - morphological tumour progress in the former irradiated region. A 10% increase in the LPFS is considered clinically relevant assuming that the LPFS rate for the proton therapy is 70%.

### Secondary objectives of the study

1. Assessment of overall survival, progression free and metastasis free survival, patterns of recurrence, local control rate and morbidity.

2. Prognostic factors, definition of patients who will benefit from the adjuvant therapy, risk group definition.

3. Plan quality (target coverage, sparing of organs at risk, integral dose).

### Study design/concept

The study is a double arm prospective randomised clinical phase III study of patients with chordomas of the skull base. Study patients are selected according to the inclusion criteria of the study protocol. After careful review of the patient reports and results of the additional examinations eligibility of a patient is determined. Patients matching the eligibility criteria and willing to participate are registered. These patients are subsequently randomised to one of the two treatment arms (arm A: carbon ion therapy, arm B: proton therapy).

Arm A: carbon ion therapy with a total target dose of 63 Gy E ± 5% to the PTV1 (s. also target definition). The PTV2 will receive a total carbon ion dose of 45 Gy E.

The patients entered in Arm B will receive proton therapy with the same target definition concept. The total proton dose will be 72 Gy E ± 5%. The PTV2 will receive a total dose of 50 to 56 Gy E in conventional fractionation (s. also dose prescription).

Accrual period for the trial will be approximately 5 years. Our study design contains interim analysis after 50% of the predicted events have occurred.

### Inclusion criteria

- Karnofsky Performance Score ≥60%

- Age >18 years and <80 years

- Informed consent signed by the patient

- Histological confirmation of chordoma with infiltration of the skull base.

### Exclusion criteria

- Inability to understand the aims of the study, no informed consent

- Prior RT of skull base region

- Other malignancies with disease-free interval < 5 years (excepting pre-cancerous lesions)

- Participation in another trial

- Pregnancy

- Simultaneous CHT or Immunotherapy.

### Risk group definition

Secondary objectives of the study will be explorative analysed also according to risk groups mentioned in Table 1.

**Table 1:** Risk group characterization

Low-risk	High-risk
Age > 30 years	Age ≤ 30 years
Male chordoma	Female chordoma
No OAR compression	Compression of OAR
Primary tumour	Recurrent tumor
Tumour size < 70 ml	Tumour size > 70 ml

### Randomisation

The randomisation will be done using the on-line randomisation tool (Randomizer.at) which is self-serve and runs exclusively on the Internet. An on-screen form with patient details will be completed. The treatment allocation will be immediately notified. The randomisation will be performed regarding treatment arms A and B. Stratification parameters include the following criteria: age (< 30, ≥ 30 years) and gender.

As this is an open-label study there will be no blinding of treatment assignment.

### Reference Committee

In order to monitor specific aspects of the current trial the Data Monitoring Committee (DMC) will be established. The work of Date Monitoring Committee will be based on the Guideline on Data Monitoring Committees EMEA/CHMP/EWP/5872/03. The DMC will be composed of independent experts in the field of radiation oncology, assessing the progress of the trial and available safety data. The mission of the DMC will be to ensure the ethical conduct of the trial and to protect the safety interests of patients in this trial.

## Radiation therapy

### Immobilisation

Patients will be immobilized using a precision head mask to ensure high repositioning accuracy of the target volume and adjacent structures for carbon ion and proton RT. Safety margins around the clinical target volume (CTV) will be determined individually for each patient. Positioning accuracy will be controlled using orthogonal x-rays or cone-beam-CTs. Set-up deviations will be corrected prior to irradiation by correction with the vector of the robotic table.

### Treatment planning examinations/Target definition

The treatment planning CT (obligate native CT, CT with contrast facultative) and MR - Examination (MRT - compulsory sequences - axial T1 post gadolinium and T2 fat saturated or Flair) will be performed in treatment position using the immobilisation device and will be co-registered. The treatment planning CT will consist of continuous 3 mm slices obtained in a stereotactic or virtual simulation set-up.

The delineation of organs at risk and target volume definition will be done on the basis of CTs and MRI scans. The PTV1 should include the GTV (entire residual tumour) and a 1-2 mm safety margin. PTV2 includes the PTV1 with individual safety margin based on surgical and histological reports, and MR-images to account for subclinical disease. The PTV2 includes the whole clivus and the prevertebral muscles down to the basis of the second cervical vertebra in any case. Normal tissue constrains will be defined as follows: eyes, optic nerves, chiasm, brainstem, spinal cord obligatory, temporal lobes, mandible, salivary glands and others facultatively. An overlap of the PTV and the OAR needs to be avoided.

### Carbon ion/Proton RT

Carbon ion RT planning is performed using the treatment planning software including biological plan optimization for carbon ions. Two to maximum four irradiation fields will be chosen. At HIT the intensity-controlled raster-scan system will be used for beam application. Considering the tolerance dose to organs at risk a dose of 63 Gy E ± 5% in 20-22 fractions for carbon ions and 72 Gy E ± 5% in 34-37 fractions for protons for chordoma patients will be prescribed to the maximum of the calculated dose distribution for the target volume (PTV1). Treatment planning aims at coverage of the PTV1 and PTV2 with the 95%-isodose line of the prescribed dose.

For carbon ion therapy dose specification is based on biologic equivalent dose because of the higher relative biologic effectiveness (RBE) of carbon ions, which differs throughout the target volume due to its dependence on various factors. RBE will be calculated for each voxel throughout the target volumes and biological optimization will be performed. The dose prescription used is related to the isoeffective dose Gy E using daily fractions of 3 Gy E and a weekly fractionation of 4-6 × 3 Gy E.

For proton therapy RBE of 1.1 will be used using daily fractions of 2 Gy E and a weekly fractionation of 4-6 × 2 Gy E.

Evaluation of DVH for the dose distribution will be performed with regards to assess plan quality. Maximum chiasm dose, maximum right and left optic nerves dose, maximum brain steam dose, median brain steam dose, maximum dose to spinal cord, median dose to spinal cord will be reported.

### Dose prescription

Arm A (carbon ion therapy):

Total dose to the PTV2 - 45 Gy E in 3 Gy E/d, 4-6 days a week, 15 fractions

Total dose to the PTV1 - 63 Gy E ± 5%, further 5-7 fractions a 3 Gy E.

Arm B (proton therapy):

Total dose to the PTV2 - 50 to 56 Gy E in 2 Gy E/d, 4-6 days a week, 28 fractions

Total dose to the PTV1 - 72 Gy E ± 5%, further 6-9 fractions a 2 Gy E.

### Dose constraints to organs at risk for both arms

Dose constraints to organs at risk (OAR) are estimated considering the experience of our institution as well as the data reported by Emami et al. [19] (Table 2).

**Table 2:** Dose constraints to organs at risk

OAR	Dose constraints
Eyes, temporal lobes, salivary glands, mandible and other	as low as possible
Optic nerves and chiasm	≤ 54 Gy
Brainstem	≤ 54 Gy with 1% of Volume allowed to receive >54 Gy, with D_max _≤ 60 Gy
Brainstem center	<50 Gy
Spinal cord	≤ 45 Gy with 1% of Volume allowed to receive >45 Gy, with D_max _≤ 50 Gy

## Organization, Workflow and Follow-up

Patient data will be collected and documented pseudonymously using electronic data processing (e.g. patient initials, date of birth and study number) at the study office at HIT. Table 3 presents a brief workflow.

**Table 3:** Workflow time table

Approval of ethics committee
↓
Diagnosis of chordoma of the skull base and completion of pre-radiation treatment examinations (biopsy, operation, staging)
↓
Informed consent of the patient and patient registration by the study office at HIT
↓
Stratification and Randomisation
↓
Arm A: carbon ion RT (63 Gy E ± 5%, fractionation 4-6 × 3.0 Gy E) Arm B: proton RT (72 Gy E ± 5%, fractionation 4-6 × 2.0 Gy E)
↓
Treatment planning for particle therapy
↓
Particle therapy
↓
Follow-up

After randomisation the study number will be assigned.

The following study data will be collected at the study office at HIT:

1. Medical reports including pathology reports and results of staging and additional pre-treatment examinations

2. Documentation of RT (dose distributions and DVH) and position checks

3. Prescribed total target dose and fraction doses, treatment interruptions >4 days

4. Toxicity data

5. Follow-up forms

6. Trial-related documentation including severe AE (CTCAE Grad ≥ 3) will be reported immediately to the study office and the principal investigator of the study

7. The date of local recurrence (MRT or CT - morphological tumour progress in the former irradiated region), distant metastases or death of the patient will be reported and documented.

Local recurrences will be confirmed radiologically and histologically whenever possible. At least two medical doctors (radiation oncologist and/or radiologist) will be required to judge of the recurrence or toxicity.

Every patient is followed for LPFS and trial-related AEs for a time period of 8 years. Furthermore, patients will be followed for survival and locoregional recurrences for a time period of 8 years after completion of the irradiation.

The first and the second follow-up examination will be performed 4-6 weeks and 3 months after completion of RT (follow-up 1 and 2). Follow-up examinations will then be scheduled after 6 months (follow-up 3), 9-12 months (follow-up 4), and then once a year for further 6 years (follow-up 5, 6 and 7). Additional visits will be scheduled as necessary. Acute toxicity is assessed at least weekly during RT and documented at the end of the RT series, 6 weeks after completion of RT and 3 months after RT. Late toxicity will be documented in regular intervals of 6 or 12 months during the observation period. All the patients will be observed for radiation specific acute and late AEs for the time of at least 8 years after irradiation. The maximum grade of toxicity will be determined for each patient.

The Common Terminology Criteria for Adverse Events V4.0 (CTCAE V.4.0) will be used to grade acute toxicity from radiation therapy. The criteria are relevant from the 8th irradiation day until day 90, i.e. until the 1st follow-up visit. Thereafter, the RTOG/EORTC Criteria of Late Effects will be utilized. All acute radiation effects will be documented on an Acute Radiation Effect-Form. In addition, the AE-Form and/or SAE-Form will be filled out. RTOG/EORTC Late Morbidity Scoring Scheme will be used to grade toxicity from radiation therapy occurring later than 90 days after its start, i.e. beyond the 1st follow-up visit. All late radiation effects will be documented on a Late Radiation Effect-Form. In addition, an AE-Form and/or SAE-Form will be filled out, if late radiation effects appear.

The follow-up examinations contain:

- Medical history

- Clinical examination

- MRI or CT of the head

- Documentation of acute side effects/AE (CTCAE V.4.0) up to 90 days after RT

- Documentation of late side effects/AE (RTOG/EORTC) after 90 days after RT

- Reporting/documentation of acute grade 3 or 4 toxicity or grade 3, 4 or 5 late effects.

### Study duration and interruption criteria

Accrual period for the trial will be approximately 5 years starting on autumn 2010. The study ends with the enrollment of planned 344 patients. Our study design contains interim analysis after observation period of 7 years. Definite assessments of LPFS, survival and for radiation specific AEs will be possible approximately 8 years after completion of radiation therapy of the last recruited patient.

The individual reasons for the study interruption are the patient dead or the withdrawing agreement of the patient to participate in the study.

With proven recurrence of disease or the development of distant metastases, the patient will be censored for our study and will be eligible for any additional appropriate therapy or inclusion in other investigative protocols, but should still be followed in order to document survival and radiation specific AEs.

The study can be early terminated under following considerations:

- high incidence of unknown AEs or increase in known AEs with the disadvantageous proportion between risk and benefits of the proposed radiation therapy;

- unacceptable high rates of SAEs;

- other reasons challenging the ethical basis;

- official decision.

All subjects who have adverse events, whether considered associated with the therapy, must be monitored to determine the outcome. The clinical course of the adverse event will be followed up according to accepted standards of medical practice, even after the end of the period of observation, until a satisfactory explanation is found or the investigator considers it medically justifiable to terminate follow-up. Should the adverse event result in death, a full pathologist's report should be supplied, if possible.

Each adverse event occurring in connection with the therapy has to be documented, independent of the cause.

## Statistical Considerations and Analysis

### Study Hypothesis

This study is designed to demonstrate that carbon ion therapy can significantly improve LPFS compared with proton therapy in the treatment of skull base chordomas. Primary objective is the comparison of the two treatment groups.

Null hypothesis H_0_: The survival functions of LPFS of the two therapies are equal, hazard ratio = 1.

Alternative hypothesis H_1_: The survival functions of LPFS of the two therapies are not equal, hazard ratio ≠ 1.

### Sample Size Determination

The sample size is calculated on the basis of the following assumptions:

• LPFS rate 5 years after radiation therapy of 70% 1) for proton therapy, hazard λ_1 _= 0.0713, and 80% for carbon ion therapy, hazard λ_2 _= 0.0446 (hazard ratio = 1.599).

• Recruitment period of 5 years, with constant accrual rate of 20% patients per year and a follow-up time of 8 years.

• One interim analysis according to a group sequential design with two stages.

Formula of Schoenfeld [20,21] used for the calculation of the number of accumulated events. For a two-sided logrank test with an overall two-sided significance level of 0.05 (including interim and final analyses) and power of 0.80, 143 events are required. The critical values and the test characteristics of the group sequential test design were calculated for an O'Brien and Fleming type alpha spending function. The computation assumes equal allocation to the two groups (n_2_/n_1 _= 1). Assuming an accrual time of 5 and a maximum observation time of 13 years a total of 319 patients is expected to yield the necessary number of events if the accrual rate is constant. Under these assumptions, the time point of the interim analysis should be 7 years after the first patient is entered. The ADDPLAN, Version 4.0 calculator was used for the sample size estimation. With the assumption that approximately 7% of the patients will be lost to follow-up a total of 344 patients will be needed to accumulate 143 events.

### Analysis Populations and General Considerations

Analysis populations will be defined as follows:

The ITT population will include all patients who are randomised, with radiation therapy administration designated according to initial randomisation, regardless of whether patients receive study treatment or receive a different treatment from that to which they were randomised. This will be the primary population for evaluating all efficacy endpoints as well as subject characteristics.

The safety population consists of all subjects who received at least one dose of irradiation administration designated according to actual study treatment received.

The PP population will consist of all subjects of the ITT population who completed the particle therapy, have at least 2 post-baseline assessments regarding the primary endpoint without pre-specified (in the statistical analysis plan), selected major protocol deviations thought to impact on efficacy analysis.

The statistical analysis will be done as soon as the database has been declared to be complete and accurate and has been locked. The details of the analysis will be laid out in the statistical analysis plan, which will be finalized and approved prior to the database lock. It has to be authorized before by the biometrician and the principle investigator. Missing values will not be replaced or imputed. For patients with incomplete follow-up, time to last follow-up date will be used as the censoring time in the analysis of time-to-event data.

### Efficacy Analysis

#### Primary efficacy analysis

LPFS is defined as the time from date of randomisation to local recurrence or death from any cause in the absence of documented local disease progression. LPFS will be censored on the date of the last tumour assessment on study for patients who do not have local tumour progression and who do not die while on study.

A log-rank test (two-sided) will be used to compare LPFS between the two treatments. The LPFS-rates will be derived from the Kaplan Meier estimate [22] and the confidence intervals will be calculated using Greenwood's formula.

#### Secondary efficacy analyses

A sensitivity analysis will be performed on the primary efficacy endpoint to assess the impact of protocol deviations using the per protocol analysis set.

A hazard ratio with 95% confidence interval for the experimental arm relative to control arm will be estimated by proportional hazard regression [23-25] with treatment, gender, age (< 30, ≥ 30 years), tumour size (< 70 ml, ≥ 70 ml), compression of OAR (yes/no) as covariates. A logistic regression analysis [26] will be carried out to identify relevant prognostic factors. Benefit from intervention (yes or no) will be defined as belonging to the patients groups (low-risk, high-risk group) which lay above the 50% percentile of LPFS.

The analyses of OS, PFS and metastasis free survival will be conducted using log-rank test. The Kaplan-Meier method will be used to obtain the estimates of the survival functions and the 95% confidence intervals will be estimated using Greenwood's formula. Covariates will not be included in the calculation.

All of these secondary analyses will be conducted at the 0.05 level of significance.

Descriptive statistics will be provided for all other secondary endpoints. Additional exploratory analyses will be used as appropriate. Generally, results will be given by summaries of the data as indicated above together with the test statistics and their associated p-values. Appropriate confidence intervals of estimates of effect will be given to quantify the degree of uncertainty of the point estimates.

### Safety Analysis

The safety analysis will be based on the safety population. The assessment of safety will be based mainly on the frequency of adverse events or serious adverse events the acute and late toxicities and on the number of laboratory values that fall outside of pre-determined ranges and/or show prominent worsening. Furthermore, the most common AEs (those occurring in at least 10% of the treatment group) will be determined. Any other information collected (e.g. severity or relatedness to study drug) will be listed as appropriate. Incidence rates and exact Pearson-Clopper 95% confidence bounds [27] of AE will be summarized.

### Interim Analysis

There is one planned interim analysis. The purpose for performing the interim analysis is to support a possible early detection of the superior therapy. In case of reaching a critical value of 2.9626 in the interim analysis the underlying therapy should neither be used for any further patients treated within the study nor for any patients outside of the study.

The interim analysis will be performed as soon as 50% of the expected events have occurred. Assuming that rate of recruitment proceeds at the rate estimated for this study the time of the interim analysis will be approximately 7 years after the first patient is entered. This time point is after the end of recruitment. For this reason the interim analysis does not have the goal to stop the study early and should not define a critical value of early stopping for futility. Independent of the result of the interim analysis the study should continue as planned with the recruited and already treated patients.

Interim statistical hypothesis tests will be performed only for the primary efficacy endpoint, LPFS. The log-rank test identical to the primary analysis will be performed at the scheduled interim analysis and compared to group sequential boundary based on the current information time. The LPFS curve estimated by the method of Kaplan-Meier will be summarized for each treatment group. The hazard ratio will also be estimated. The interim safety report will review all aspects of the data collected for each subject. Other important additional factors that reflect the integrity of the protocol will be addressed in the interim report.

A group sequential design with an O'Brien and Fleming alpha spending function is used to control the type I error. The nominal one-sided significance level for the interim analysis determined by using the O'Brien-Fleming stopping rule is 0.0015.

## Ethical and legal aspects

The study plan has been submitted to the ethics committee of the Medical faculty Heidelberg for approval. The written approval by the ethics committee has been already obtained. Additionally, we received the positive vote of Bundesamt für Strahlenschutz for this study.

Prior to initiation of the treatment, patients will be informed about the goals of the trial in a conversation. Potential advantages and treatment related risks will be discussed with the patient. A written consent will be obtained and documented. The patient will be informed about the possibility to withdraw his agreement to participate in the study at any time without giving reasons. The responsible physician will inform the patient in his own language if possible and keep a record of this in the patient chart. The informed consent form will be signed. In case of cancellation the patient will be offered standard therapy.

The coordinating investigator has to subscribe to an insurance policy covering, in its terms and provisions, its legal liability for injuries caused to participating persons and arising out of this research performed strictly in accordance with the scientific protocol as well as with applicable law and professional standards.

Any impairment of health which might occur in consequence of trial participation must be notified to the insurance company. The subject is responsible for notification. The insured person will agree with all appropriate measures serving for clarification of the cause and the extent of damage as well as the reduction of damage.

During the conduct of the trial, the subject must not undergo other clinical treatment except for cases of emergency. The subject is bound to inform the investigator immediately about any adverse events and additional drugs taken. The terms and conditions of the insurance will be handed out to the subject on request.

The insurance company has to be informed about all amendments that could affect subjects' safety.

## Discussion

This study is designed as a prospective monocentric randomised phase III trial. The trial will be carried out at HIT centre. Proton therapy is the gold standard in the treatment of skull base chordomas. However, high-LET beams such as carbon ions theoretically offer biologic advantages by enhanced biologic effectiveness in slow-growing tumours. Up until now it was impossible to compare two different particle therapies, i.e. proton and carbon ion therapy directly with each other at the same institution. The aim of this study is to find out, whether the biological advantages of carbon ion therapy mentioned above can also be clinically confirmed.

## Competing interests

The authors declare that they have no competing interests.

## Authors' contributions

AVN, IKS, MWM, ADJ, SEC, and JD have developed the study concept. AVN, MWM, IKS, ADJ, SEC and JD wrote the study protocol and obtained ethics approval. IKS was responsible for statistical considerations. AVN, ADJ, MWM, SEC and JD will provide patient care. AVN, IKS, MWM, ADJ, SEC and JD will implement the protocol and oversee collection of the data. All authors contributed to and approved the final manuscript.

## Pre-publication history

The pre-publication history for this paper can be accessed here:

http://www.biomedcentral.com/1471-2407/10/607/prepub
